# Social prescribing for socially isolated older adults in rural Japan: a qualitative case study

**DOI:** 10.3389/fpubh.2025.1659713

**Published:** 2025-10-16

**Authors:** Naho Ota, Mayu Ebihara, Mizuki Aoki, Atsushi Iwasawa, Teiichiro Yamazaki, Songee Jung, Sachiko Makabe, Mary Lynch, Charlotte Rothwell, Jan Illing, Kyoko Nomura

**Affiliations:** ^1^School of Medicine, Akita University, Akita, Japan; ^2^Department of Environmental Health Science and Public Health, Akita University Graduate School of Medicine, Akita, Japan; ^3^Department of Nursing, Akita University Graduate School of Health Sciences, Akita, Japan; ^4^Faculty of Nursing and Midwifery, Royal College of Surgeons in Ireland, University of Medicine, and Health Sciences, Dublin, Ireland; ^5^Population Health Sciences Institute, Newcastle University, Newcastle upon Tyne, United Kingdom; ^6^Health Professions Education Centre, Royal College of Surgeons in Ireland, RCSI University of Medicine and Health Sciences, Dublin, Ireland

**Keywords:** social prescribing, link workers, older adults, social isolation, general practitioners, qualitative research

## Abstract

**Introduction:**

Social prescribing (SP) has not yet been officially introduced in Japan. This qualitative case study aimed to identify the challenges and facilitating factors in the implementation of SP among the socially isolated older population in Akita Prefecture, Japan, based on the perspectives of general practitioners (GP), link workers (LW), and patients.

**Method:**

We conducted a qualitative case study using semi-structured interviews and Braun & Clarke-informed thematic analysis in seven medical districts in Akita, Japan, with GP (*n* = 7), LW (*n* = 10), and older patients (*n* = 4).

**Results:**

Participants (GP and LW) emphasized that SP needed to be tailored to individual needs and that LW played a vital role as social resources in sparsely populated rural communities. The project was publicly funded; participants emphasized that, in the absence of financial support, intrinsic motivation would be important to sustaining implementation. Both groups raised concerns about ensuring LW’s competencies, the accessibility and cost of community resources, and the limited availability of such resources in rural areas. Patients highlighted that the effectiveness of SP varied by personal characteristics, and that transport barriers significantly restricted participation, highlighting the need for local support to mitigate this challenge. Establishing patients’ trust in LW and GP, along with effective communication, was viewed as essential for identifying and addressing patient-level barriers.

**Conclusion:**

In rural, resource-constrained settings such as Akita, successful SP depends on tailoring to individual needs, ensuring LW competence, and addressing transport barriers. These findings suggest that future policies should focus on sustainable funding for LW, integration with existing health and welfare systems, and mobility solutions.

## 1 Introduction

Social Prescribing (SP) is defined by the World Health Organization as a means of connecting patients to nonclinical community services to improve health and well-being (1). Loneliness and social isolation negatively affect well-being, subjective health, cognitive function, depression, and all-cause mortality, and are increasingly recognized as global public health issues requiring preventive approaches (2–7). SP has therefore gained particular attention in Western countries as a strategy to address these challenges, alongside other social determinants of health (SDH) (8–11).

Japan faces an unprecedented demographic challenge, with 29% of the population aged ≥65 years, projected to reach 38.4% by 2065 (12). In 2021, the Japanese government’s Basic Policies for Economic and Fiscal Management and Reform first identified SP as a preventive measure against isolation and loneliness (13). Between 2021 and 2023, pilot projects were launched in several prefectures, including Akita Prefecture, which has Japan’s highest proportion of older adults and severe winter isolation, as one of the pilot sites.

Despite growing interest, Japan lacks a formal infrastructure or public funding framework for SP, and little is known about how it can be implemented in resource-limited rural prefectures. Therefore, this study examined the perspectives of general practitioners (GP), link workers (LW), and socially isolated older adults in Akita Prefecture to identify key challenges and facilitating factors for implementing SP.

## 2 Methods

### 2.1 Study design

This study employed a qualitative case study design, with the case defined as a taskforce-led SP pilot across seven medical districts in Akita Prefecture. While this approach has inherent limitations in that its findings are difficult to directly generalize and may be subject to researcher bias (14), it was chosen for its ability to explore in-depth complex, context-dependent phenomena (14–16). This enabled a comprehensive understanding of the underlying processes and mechanisms that quantitative methods cannot sufficiently capture.

In 2021, a taskforce was formed in Akita Prefecture to conduct a pilot project on SP. The taskforce included the Akita Prefecture Government, the Akita Medical Association, the Department of Environmental Health Science and Public Health at Akita University, the Akita Health Insurance Council, and other relevant organizations. The taskforce first included five GP; after 2022, three additional GP were added to the task force to test its effectiveness in a larger number of subjects. The taskforce members, including GP, recruited 18 LW through their own social networks, including their clinical and relevant workplaces. GP referred LW to patients, and the LW visited patients’ homes and identified patients’ conditions using an assessment sheet.

The assessment sheet (Supplementary material 1) used in this study was newly created by referring to existing scales (Hasegawa Dementia Scale (17), UCLA Loneliness Scale (18)), questions from publicly available websites (19), and original questions developed specifically for the purpose of this study. To ensure its validity and relevance, the sheet was iteratively revised over the three-year project based on feedback from experts, including a professor from the Department of Public Health, GP and LW involved in the study. The assessment sheet developed for the present study included questions about living alone (20), medical or nursing care requirements (21), shut-in (socially isolated) (18, 22), economic hardship (23–25), mental health status (21, 26), environmental hygiene (known as a trigger for dementia) (27–29), someone asking for help (30), and dementia (a reason for social isolation) (17, 31, 32). Based on the assessment sheet, LW referred patients to local social resources.

**Figure 1:**
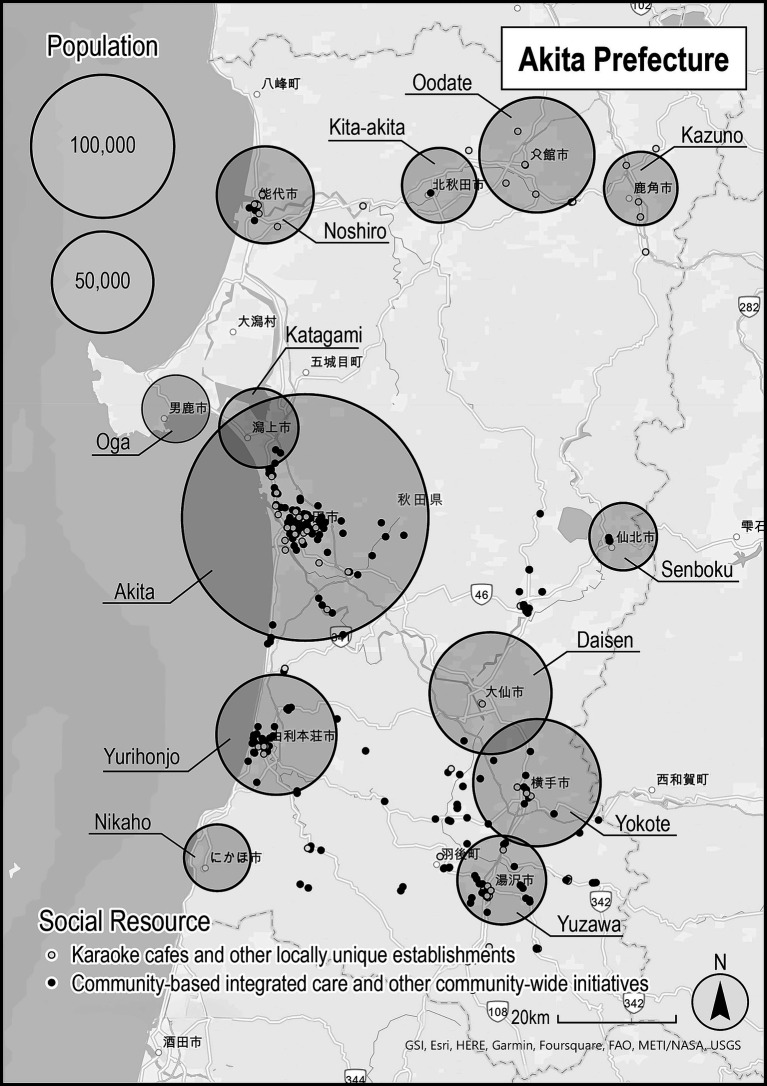
Distribution of social resources and population in Akita. Data sources: Map—ArcGIS Pro (Esri, United States); Social resources map created by the Akita Social Prescribing Taskforce in 2021; Population—Akita Prefecture, Mino Kuni Akita Net (https://www.pref.akita.lg.jp/pages/archive/2078), 2021 data. White dots, Karaoke cafes and other locally unique establishments. Black dots, Community-based integrated care and other community-wide initiatives.

From 2021 to 2023, GP selected 47 patients. Among the 47 patients, only 22 had access to social resources. The social resources defined in this study include exercise groups, tea parties, knitting groups, volunteer groups, public assistance, and nursing care insurance. Figure 1 shows the social resources in Akita Prefecture, available in 2021, and represented by dots. This map was created within the taskforce, using ArcGIS Pro (Esri, United States) and only publicly available social resource information. The map shows that social resources in Akita Prefecture are concentrated in large cities and are sparse in other areas.

### 2.2 Study participants and study framework

The attributes of the participants are shown in Table 1. Participants were purposively selected to include a diverse range of characteristics and backgrounds. The study included seven GP (all male, mean age: 57.1 ± 10.9 years) and 10 LW (male: 1, female: 9, mean age: 63.0 ± 10.9 years) from seven medical districts of Akita Prefecture. We directly invited GP and LW to participate through existing connections within the task force. Some LW joined the study at the request of their affiliated GP.

**Table 1:** Attributes of general practioners, link workers and patients.

Participant	Age	Gender	Department
General practitioner	67	M	Surgery
	43	M	Internal Medicine
	54	M	Internal Medicine
	46	M	Surgery
	54	M	Surgery/Internal Medicine
	64	M	Internal Medicine
	72	M	Urology
Link worker	73	F	Former Public Health Nurse
	48	F	Public Health Nurse
	62	F	Nurse
	68	F	Nurse
	76	F	Former Public Health Nurse
	70	F	Former Public Health Nurse
	71	F	Former Public Health Nurse
	61	F	Nurse
	58	M	Social Welfare Council
	43	F	Social Worker
Patient	75	F	Not employed
	89	F	Not employed
	76	M	Not employed
	65	F	Not employed

We also asked participating GP and LW to assist with the recruitment of patients. Using a research brochure that explained the concept of SP and provided an overview of this study, they recruited individuals who met the following criteria: (1) were cooperative and (2) were facing or at high risk of social isolation (e.g., older caregivers or older adults living alone). Of the 47 patients enrolled in the pilot project, only four were interviewed (male: 1, female: 3, mean age: 76.3 ± 9.8 years), as most were excluded due to cognitive impairment, frailty, or the perceived burden of participation. Seasonal factors, such as heavy snowfall and limited transport in rural Akita, along with COVID-19 restrictions, further limited patients’ availability and researcher visits. Consequently, the patients’ sample was small, and the transferability of findings should be interpreted with caution.

### 2.3 Data collection

One-on-one semi-structured interviews were conducted between Dec 7, 2021, and Oct 8, 2023. Separate interview guidelines were developed for GP, LW, and patients (Supplementary material 2). The interviews were conducted via online (Zoom Communications, Inc.) for GP and LW; two patient interviews were conducted by telephone and two in person. Prior to the interviews, seven medical students and one faculty member were trained to perform equally by two faculty members through role-playing. A verbatim transcript was created from the audio data recorded by the researchers. Data collection continued until no substantially new codes or themes emerged, with saturation reached after 17 interviews with GP and LW. Four patients’ interviews were included to provide complementary perspectives, although further recruitment was limited by frailty and cognitive impairment among the eligible patient population. Thus, the final sample was considered adequate to capture variation across professional and patients’ experiences.

### 2.4 Data analysis

A team of seven medical students, one graduate student, and three faculty members conducted a thematic analysis following Braun and Clarke’s six-phase framework (33): familiarization, coding, theme development, review, definition, and reporting. Each interview transcript was assigned to a single team member for initial coding using MAXQDA Analytics Pro 2022 (Release 22.2.0), focusing on the perspectives of GP, LW, and patients regarding the implementation of SP. Any questions or disagreements regarding codes were resolved through team discussion and consensus-building. The first author then organized similar codes into subcategories, categories, and overarching themes. A subsequent team meeting was held to review and refine the categories and themes based on each researcher’s perspective, ensuring that coding and interpretation did not rely on a single viewpoint and enhancing analytical rigor. At this stage, the team confirmed that no additional categories were emerging, supporting the conclusion that data saturation had been reached. Finally, the results were shared with all GP and a subset of LW to verify that the findings accurately reflected participants’ perspectives (data triangulation). GP and LW’s perspectives, which were similar, were presented together, whereas patients’ perspectives were presented separately due to the emergence of distinct categories.

### 2.5 Trustworthiness

To enhance the rigor of this qualitative case study, we applied Lincoln and Guba’s four criteria of trustworthiness: credibility, dependability, confirmability, and transferability (34). Credibility was supported through investigator triangulation and member checking with all GP and selected LW, ensuring that interpretations accurately reflected participants’ perspectives. Dependability was ensured by discussing and resolving discrepancies among coders within the team, maintaining consistency in analytic procedures. Confirmability was strengthened by systematically documenting and sharing data and analytic procedures, and by storing codes and transcripts in MAXQDA to ensure auditability. Transferability was promoted by including participants with diverse roles, occupations, ages, and sexes, and by providing a detailed description of the rural context in Akita Prefecture.

### 2.6 Ethical considerations

The study protocol was approved by the Ethics Committee of the Akita Medical GP Association (approval No. 52, October 24, 2023). Informed consent was obtained orally from all GP, LW, and patients prior to participation. The audio data was promised to be deleted after the completion of the study.

## 3 Results

Demographic characteristics of all participants can be shown in Table 1.

### 3.1 Perspectives of GP and LW

The average interview duration was 39 min and 43 s for GP (*n* = 7, all male, mean age: 57.1 ± 10.9 years) and 38 min and 53 s for LW (*n* = 10, male: 1, female: 9, mean age: 63.0 ± 10.9 years). We identified four major themes through thematic analysis. These themes, their categories, frequencies, and representative quotes are summarized in Table 2, while the following sections describe them in detail.

**Table 2:** General practitioners and link workers’ perspectives: themes, categories, exemplar quotes, and frequency (n/17).

Theme	Category	Exemplar quotes
1. Social prescribing (SP) tailored to patients’ individual needs	1.1 Patients’ various settings and barriers (12/17)	“The patient had difficulty walking, was afraid of falling, and couldn’t go outside alone.” (LW, female)
	1.2 Patients’ motivation (6/17)	“Some patients are not very social but want to connect with others and find something to do.” (LW, female)
	1.3 Importance of collecting background and needs information (17/17)	“To find suitable social resources, we ask patients about their past activities and hobbies.” (GP, male)
2. Link workers (LW) could be a social resource	2.1 Patients’ good partners (11/17)	“In rural areas, LW acts as social resource, comforting patients by listening to their concerns.” (GP, male)
	2.2 Trust between LW and patient (5/17)	“Building trust and introducing social resources takes time but helps patient access.” (LW, female)
	2.3 Extensive knowledge of social resources (5/17)	“LW experience in discharge coordination helps as they know local resources well.” (GP, male)
	2.4 Multidisciplinary collaboration (8/17)	“LW cannot handle all patients’ issues alone; we collaborate with GP and institutions, especially for financial matters.” (LW, female)
	2.5 Acting as contact points for social resources (6/17)	“For those not on long-term care insurance, being connected to services is low; an intermediary is essential.” (LW, female)
3. GP and LW need motivation for implementing SP when no public subsidies exist	3.1 Ongoing support for patients (5/17)	“Older patients’ needs vary by physical and medical conditions; continuous support is needed.” (LW, female)
	3.2 Maintaining motivation (13/17)	“SP aims to improve community well-being and should be encouraged in Japan.” (GP, male)
4. How do we secure the quality of LW, the numbers of social resources, access and cost?	4.1 Independent LW (8/17)	“It is important to choose LW who knows local communities and resources.” (GP, male)
	4.2 Communication for multidisciplinary collaboration (5/17)	“Patients’ communication often had to go through the GP’s clinic, leading to some misunderstandings.”(LW, female)
	4.3 Securing appropriate social resources (14/17)	“Patients bearing some costs help sustain social resources.” (LW, male)
	4.4 Patients’ transportation and costs (14/17)	“Transportation is a major bottleneck; patients may struggle to attend appointments.” (GP, male)

#### 3.1.1 SP tailored to patients’ individual needs

Patients’ diverse backgrounds and health conditions influenced their engagement with SP. Three categories emerged. First, patients experienced different settings and barriers to engaging with SP (i.e., “Patients’ various settings and barriers” to SP, mentioned by 12 out of 17 participants). Second, these differences affected their motivation to participate in SP (i.e., “Patients’ motivation” for SP, mentioned by 6 out of 17 participants). Finally, participants emphasized the importance of collecting detailed information about patients’ backgrounds and needs to ensure appropriate referrals to social resources (i.e., “Importance of collecting background and needs information,” mentioned by 17 out of 17 participants).

#### 3.1.2 LW could be a social resource

LW provided emotional support, built trust, and coordinated with multiple stakeholders to facilitate patient access to social resources. There were five categories. LW was found to play an important role as social resources. The LW becomes the patient’s talking partner (i.e., “Patients’ good partners,” mentioned by 11 out of 17 participants) and encourages the participation of social resources by building a trusting relationship with patients (i.e., “Trust between LW and patient,” mentioned by 5 out of 17 participants). They worked as LW based on their extensive knowledge of social resources obtained from their usual job activities (i.e., “Extensive knowledge of social resources,” mentioned by 5 out of 17 participants), but they also collaborated with practitioners, other medical professionals, government officials, and other multidisciplinary professionals to obtain additional background information on social resources (i.e., “Multidisciplinary collaboration,” mentioned by 8 out of 17 participants). They also served as points of contact for introducing social resources (i.e., “Acting as contact points for social resources,” mentioned by 6 out of 17 participants).

#### 3.1.3 GP and LW need motivation for implementing SP when no public subsidies exist

GP and LW emphasized that ongoing support and commitment are crucial despite time constraints. There were two categories: GP and LW indicated that ongoing patient involvement, support, and time are essential for SP to be successful (i.e., “Ongoing support for patients,” mentioned by 5 out of 17 participants). GP and LW sometimes found it time consuming to refer patients to SP in addition to their regular work. Despite these difficulties, they understood the importance of implementing SP for socially isolated patients (i.e., “Maintaining motivation,” mentioned by 13 out of 17 participants).

#### 3.1.4 How do we secure the quality of LW, the numbers of social resources, access and cost?

Participants identified training, coordination, resource availability, and transportation as key challenges. There were four categories. In addition to clarifying the position of LW, LW candidates need to be trained to take on the role of LW (i.e., “Independent LW,” mentioned by 8 out of 17 participants). LW needed help communicating and coordinating schedules with multiple professions, patients, and key persons (i.e., “Communication for multidisciplinary collaboration,” mentioned by 5 out of 17 participants). In this regard, one GP noted that this profession requires an understanding of confidentiality regarding patients’ privacy. Due to the shortages in social resources in rural depopulated areas like Akita, it is questionable whether available and suitable social resources for patients exist (i.e., “Securing appropriate social resources,” mentioned by 14 out of 17 participants). It is also very important to understand how patients can secure transportation means and costs from their homes to access such resources, as there is no or very limited transportation available to this population (i.e., “Patients’ transportation and costs” to participate in social resources, mentioned by 14 out of 17 participants).

### 3.2 Perspectives of patients

The average interview duration was 53 min and 4 s for patients (*n* = 4, male: 1, female: 3, mean age: 76.3 ± 9.8 years). We identified three major themes based on patients’ perspectives. These themes, their categories, frequencies, and representative quotes are summarized in Table 3, while the following sections describe them in detail.

**Table 3:** Patients’ perspectives: themes, categories, exemplar quotes, and frequency (n/4).

Theme	Category	Exemplar Quote
1. Individuals’ characteristics of the recipients	1.1 Patients’ own health and various environmental factors (4/4)	“My knees hurt, so when I walk, I can only walk a short distance in front of my house or about 5–6 min to a friend’s house.” (Female)
	1.2 Vitality from conversations with others (2/4)	“It’s a nice change of pace… if I go and do not talk to anyone, then I do not see the point of coming.” (Male)
	1.3 Social resources that they agree with (3/4)	“If it’s a (social resource) that suits me, I might participate.” (Female)
2. Struggle with transportation	2.1 Returning a driver’s license (1/4)	“I also gave up my driver’s license, so I must use the pick-up and drop-off vehicles.” (Male)
	2.2 Difficulty of moving in winter (2/4)	“The heavy snow made it impossible to drive, so I was unable to attend the exercise class the LW recommended.”(Female)
	2.3 Local support (public transport, neighbors) (4/4)	“I asked neighbors to pick me up and drop me off and actively used public transportation and courtesy cars.” (Female)
3. Establishing patients’ trust of link workers (LW) and general practitioners (GP)	3.1 Confidence in healthcare providers (4/4)	“My GP is a kind person who listens carefully to me and is easy to talk to.”(Male)
	3.2 Good communication with LW (3/4)	“LW is someone who listens to what I want to talk about. (Male)”

#### 3.2.1 Individuals’ characteristics of the recipients

Patients’ health conditions, living arrangements, and personal preferences influenced their engagement with SP. There were three categories: four patients who participated in SP had their own health issues and various concerns in their daily lives, such as living alone as older adults or living as older couples. (i.e., “Patients own health and various environmental factors,” mentioned by 4 out of 4 participants). Patients stated that they felt invigorated by talking with LW, people they met with social resources, family, and friends (i.e., “Vitality from conversations with others,” mentioned by 2 out of 4 participants). To participate in social resources, patients need to be healthy and in a community that suits them (i.e., “Social resources that they agree with,” mentioned by 3 out of 4 participants).

#### 3.2.2 Struggle with transportation

Limited transportation options, especially for older patients, created barriers to accessing social resources. There were three categories: Many of the patients had given up their driver’s licenses due to illness or advanced age (i.e., “Returning a driver’s license,” mentioned by 1 out of 4 participants), making it difficult for them to get around, especially in the winter when it snows (i.e., “Difficulty of moving in winter,” mentioned by 2 out of 4 participants). However, they used mobile vending services to buy groceries, asked neighbors to pick them up and drop them off, and actively used public transportation and courtesy cars (i.e., “Local support,” mentioned by 4 out of 4 participants).

#### 3.2.3 Establishing patients’ trust of LW and GP

Trust in healthcare providers and good communication with LW facilitated engagement with SP. There were two categories: LW and patients had a good relationship with the four patients we interviewed. Patients had trust in healthcare providers and participated in SP (i.e., “Confidence in healthcare providers,” mentioned by 4 out of 4 participants). They said that even if they were not participating in social resources, they felt more comfortable talking with their LW about daily issues or concerns (i.e., “Good communication with LW,” mentioned by 3 out of 4 participants).

## 4 Discussion

GP and LW’s perspectives were similar and revealed four themes, while patients’ perspectives yielded three themes. GP and LW reported that SP needs to be individualized for each patient, and patient interviews similarly revealed that differences in patients’ personal characteristics and physical conditions often made accessing social resources challenging. Moreover, GP and LW suggested that LW themselves could function as social resources for patients. All four patients reported that their LW served as a trusted conversational partner who alleviated loneliness. This illustrates an intrinsic motivation to reduce loneliness which appeared to be associated with SP uptake when matched with a reliable human connection. This may further suggest that human relationships and interaction are not merely due to the availability of physical resources but could also be a key driver for engagement. At the same time, patients without private vehicles relied on public transport or local pick-up services, but limited availability of these options constrained access even when motivation to engage was present. Even those with cars were sometimes unable to travel in winter due to heavy snow, indicating that seasonal factors introduced unpredictable barriers that eroded motivation over time. Both GP and LW identified transport challenges as an important factor that may hinder the sustainability of SP. In addition, GP and LW raised concerns about the quality and workload of LW, the adequacy of available social resources relative to patients’ needs, and associated costs. These factors together may highlight a mechanism whereby motivation alone was insufficient to ensure uptake when appropriate, accessible resources were lacking.

This tailored support may be key to SP success, as shown in previous studies (35–39). In addition, although it may depend on recipient characteristics and individual human relationship difficulties, many of the patients perceived the benefits of social interactions, such as conversations with their friends (40) or LW as positive (25). For example, LW was able to become a social resource by presenting themselves as simple listeners to patients’ worries and concerns. Many previous studies have also suggested that trusting relationships between LW and patients may represent central elements in achieving well-being and are the key to successful linkages to suitable services (28, 41, 42). In addition, such active listening provided by LW met patients’ needs in underserved areas, such as Akita, where transportation in winter is impeded due to heavy snowfall.

Nevertheless, as previous studies have shown (41, 43), the availability of social resources, such as local communities and transportation, appears to be an important factor for SP implementation. As a possible solution, LW could collaborate with patients to create social resources concurrently with SP. As suggested by previous evidence (27), SP from a medical perspective often centers on concepts such as person-centeredness, empowerment, and co-production. One such study appeared to show how a few older recipients of SP were empowered by the LW and subsequently built social resources collaboratively within limited regions (27). Furthermore, another study has indicated that the goal of SP can be to increase recipients’ self-confidence and enable them to make their own plans for social independence, such as forming walking groups, community cafés, or cooking groups (42). In our study, there was an example of LW who planned to open a coffee shop. These activities are not only empowering but can also be a means of co-producing plans and social advancement in the current aging population (5) Such activities may be empowering and could also be a means of co-producing plans and social advancement in the current aging population (11). These activities require long-term involvement and support from LWs, as well as their experience in community development (44). Future studies should explore Information and Communication Technology (ICT) applications to social resources in underserved areas where both social and personnel resources are scarce (45). Online services may also be an area for exploration, especially in the winter months when many older adults have difficulty getting around. Emerging evidence suggests that meeting people virtually may have similar positive effects to meeting them in person (46–48). Regarding transportation, the use of automated vehicles in rural areas is considered potentially able to improve the quality of rural public transportation (49). While ICT solutions and autonomous transport hold long-term potential, their feasibility is constrained by high costs (50) and the necessity of infrastructure development (51). Additionally, low digital literacy among the older adults (52) and concerns about data governance and privacy (53) are also challenges. As immediate, low-cost alternatives, interventions such as telephone befriending programs (54), the development of nature-based activity programs with demonstrated mental health benefits (55), and demand-responsive transportation and shared taxis (56) could provide accessible support for socially isolated older adults in rural areas like Akita.

In addition, SP that involves one-on-one support between LW and recipients can be extremely time-consuming (52), and has been criticized for its low cost-effectiveness and the concern that it may only benefit a limited group of people (57–59). While GP and LW recognize the benefits and are motivated to engage in such activities, carrying them out on a voluntary basis in addition to their regular duties places a considerable burden on them (60–63). Therefore, it is important to implement SP as efficiently as possible while utilizing existing systems.

Firstly, the points of contact for SP may warrant consideration. In the United Kingdom, where SP originated, GP-led SP was introduced in the government’s long-term plan in 2019 (64), under which GP is reimbursed on a per capita basis, with GP receiving payment based on the number of patients they manage. This structure facilitates the establishment of long-term patient relationships and emphasizes reducing the frequency of visits and alleviating staff workload, thereby enabling greater focus on preventive care. Consequently, GP-led SP has become prevalent in the UK; however, some challenges exist owing to the new official started (64). In Japan, patients have the autonomy to visit any medical facility of their choice, resulting in GP not overseeing all patients and a lack of continuity of care. In addition to medical insurance, Japan also has a long-term care insurance system that caters to older adults who may not need medical care but need welfare services, specific health checkups, and specific health guidance for people aged 40–74. Like Japan, South Korea’s healthcare system is not GP-managed, although local primary care clinics similarly play a GP-like role in providing first-contact care. During the COVID-19 pandemic, a community-based SP pilot in a rural South Korean community statistically confirmed a reduction in loneliness and depression among older adults, as well as an increase in their sense of social participation and self-esteem (65). This model was not GP-led; rather, it leveraged local community resources. Therefore, it is desirable to establish multiple points of contact to implement SP effectively. In this study, a questionnaire was designed for the early detection of social isolation in social and medical settings.

Secondly, in a nation without LW, the key issue is who fulfills this role (30). For example, similar challenges have been reported in Romania, where the formal roles of LW and the structures for SP are not well-established (66). In the UK, LW is employed by organizations in the voluntary sector and are either commissioned to work for the national health service (NHS) or are hired directly by the NHS (67). In Japan, there are already roles fulfilling health counseling for older adults in the community, such as in large hospitals and long-term care welfare facilities. The problem, however, is not only the lack of these roles and the consistent nature of the work, but also the degree to which patients can be connected to social resources. In urban areas, where social resources are plentiful, existing positions may easily connect patients to social resources. However, in Akita Prefecture, where social resources are scarce, as in this study, LW are required not only to refer patients to social resources but also to provide transportation (68, 69) and create new social resources to support them in getting to social resources. In this regard, it may be possible to divide the functions of LW by strengthening multidisciplinary collaborations. To implement SP, it is a challenge to secure a stable financial foundation. For instance, it is a matter for consideration how to set an appropriate remuneration for LW and related multidisciplinary teams. It is also thought to be necessary to consider patient payments in addition to the framework of the insurance system. Some GP and LW who participated in this study expressed the view that social resources need to be something patients feel are worth paying for.

One of the main strengths of this study is the triangulation of perspectives, incorporating GP, LW, and patients, which allowed for a comprehensive understanding of factors affecting the implementation of SP. In addition, this study provides Japan-specific insights into facilitators and barriers, offering valuable information for promoting SP in underpopulated areas with limited social resources. The study also highlights specific and actionable themes, such as transportation challenges, workforce limitations, and social resource mapping. These findings may inform policy-making and practical implementation efforts aimed at establishing SP in local communities.

This study has several limitations. Firstly, only four patients were interviewed, which limits the transferability of patients’ perspectives. In addition, there is a possibility of selection bias, as participating patients were mainly recruited via their GP, and participating GP and LW were likely more motivated to take part in SP intervention. Future studies should recruit larger and more diverse patients’ samples through multiple channels, independent of GP networks, to reduce this bias. Secondly, responses from GP and LW may have been influenced by social desirability bias and the structure of the interview guides, and future work could incorporate anonymous surveys or mixed methods to mitigate these effects. Thirdly, there is a possibility of selection bias, as participating GP and LW were likely more motivated toward SP, and patients were mainly recruited via their GP, which may have affected who was invited and agreed to participate. Finally, the study was conducted in a single rural prefecture (Akita), and findings may not be directly generalizable to urban or other regional contexts in Japan; comparative studies across multiple settings are warranted.

## 5 Conclusion

In rural, resource-constrained settings such as Akita, successful SP depends on tailoring to individual needs, ensuring LW competence, and addressing transport barriers. These findings suggest that future policies should focus on sustainable funding for LW, integration with existing health and welfare systems, and mobility solutions.

## Data Availability

The original contributions presented in the study are included in the article/Supplementary material, further inquiries can be directed to the corresponding author.
