# Supplementary material for: Social prescribing for socially isolated older adults in rural Japan: a qualitative case study
Source: Front Public Health. 2025 Oct 16;13:1659713. doi: 10.3389/fpubh.2025.1659713 (PMC12571642; doi:10.3389/fpubh.2025.1659713)
Supplement: Supplementary file 2 [file Table_2.docx]

Supplementary Material

# Supplementary material 2

Supplementary material 2 contains separate interview guides for one-on-one semi-structured interviews, focusing on the perspectives of GP, LW, and patients regarding the implementation of SP.

| Topic | General Practitioner (GP) | Link Worker (LW) | Patient |
| --- | --- | --- | --- |
| 1. Background | How many patients were connected to social prescribing (SP) this time? Please tell me about each patient. | Could you tell me about the specific steps a patient takes to participate in SP? | How do you understand the reason you were referred to LW? |
| 2. Facilitating & Inhibiting Factors | In cases where SP was successful, what was the key factor? | For cases where SP was successful and those that weren't, what do you think made the difference? | Were you introduced to any social resources by your GP or LW? |
| 3. Difficulties | What difficulties did you encounter in practicing SP? | What difficulties did you encounter when providing SP support? | Did you want to participate in social resources? Why or why not? |
| 4. Collaboration | How did you collaborate with LW for this project? | What kind of support did your GP provide? | - |
| 5. Social Resources & Transportation | What kind of transportation did your patients use, and how did they pay for the costs? | What kind of transportation did your patients use, and how did they pay for the costs? | What kind of social resources would you like to participate in? (Frequency, location, cost, transportation) |
| 6. Time and Burden | How much time did you spend on SP? In terms of time and cost, do you think you can continue it in the future? | Do you think you can continue this work alongside your current job in the future? | - |
| 7. Relationships | - | - | Was there anything your GP did that helped you or made you happy? Conversely, was there anything you wish they had done? |
|  |  |  | Was there anything LW did that helped you? Conversely, was there anything you wish they had done? |
| 8. Needed Support | What kind of support do you think you need to continue SP in the future? | What kind of support do you think you need to continue SP in the future? | - |
| 9. Summary | What are your final impressions of SP as a whole? | How do you feel about your experience with this SP activity? | Please share your final opinion on SP as a whole. |
